# Heart rate variability in people with visual disability: Study Protocol

**DOI:** 10.1097/MD.0000000000017656

**Published:** 2019-11-15

**Authors:** Renaldo D. Moreno, Luiz C. Abreu, Mauro J.D. Morais, Fabiano S. Oliveira, Italla M.P. Bezerra, Vitor E. Valenti, Monica A. Sato

**Affiliations:** a Universidade Federal do Acre, UFAC, Rio Brancro; b Setor de Pos-graduação, Pesquisa e Inovação; c Laboratório de Delineamento de Estudos e Escrita Cientifica, Faculdade de Medicina do ABC, Centro Universitario Saude ABC, Santo Andre, SP; d Programa de Pos-graduação em Politicas Publicas e Desenvolvimento Local, Escola Superior de Ciencias da Santa Casa de Misericordia de Vitoria, EMESCAM, Vitoria; e Laboratório Multidisciplinar de estudos e Escrita Científica das Ciências da Saúde - LAMEECCS, UFAC, Acre, Brazil; f Graduate Entry Medical School, University of Limerick, Limerick, Ireland; g Universidade Estadual Paulista (UNESP), Centro de Estudo do Sistema Nervoso Autonomo, Marilia; h Department Morphology and Physiology, Faculdade de Medicina do ABC, Centro Universitario Saude ABC, Santo Andre, SP, Brazil.

**Keywords:** autonomic nervous system, blindness, heart rate, visual impairment

## Abstract

**Introduction:**

People with visual impairment (VI) have loss of vision that causes impact on their daily living activities. Synonymous of VI are blindness, low vision, subnormal vision, visual incapacity, although there are peculiarities among them. The autonomic nervous system (ANS) provides the body with dynamic adaptation, moment by moment, according to changes in the internal and/or external body environment. As VI is an adverse condition, it is expected to be associated with changes in systemic autonomic activity, such as heart rate (HR) variability.

**Objective:**

To analyze the blindness stress by monitoring the activity of the ANS in the heart in subjects submitted acutely to low vision and also in subjects with chronic visual deficiency.

**Method:**

This is a randomized trial experimental study. In this clinical trial, initially, patients will undergo an ophthalmologic medical evaluation, along with monitoring of HR and systolic blood pressure /diastolic blood pressure. Volunteers with normal vision (Group i); and people with VI (Group ii) will be evaluated, all of them inhabitants of Rio Branco City, capital of Acre State, Brazilian Amazon. The intervention will consist of simulating blindness by sealing both eyes of each participant with good eyesight, using a sleep mask and allowing maximum occlusion for 45 minutes, split into 3 periods of 15 minutes each. Still blindfolded, participants will be requested to perform different tasks as walking, serve themselves water and/or cookies, and engaging in playful-pedagogical activity. Identical procedure will be done with the group with VI. The HR will be recorded by the Polar RS800 HR monitor. All findings with a value of *P* < .05 will be considered statistically significant. As a risk measure the odds ratio will be calculated, adjusted, and not adjusted with their respective 95% confidence intervals. The odds ratio = 1 of lowest risk for the outcome of interest will be considered as the base category for each independent variable.

**Ethics and dissemination:**

This study will be carried out in accordance with the guidelines that regulate human research in Resolution No. 466/12 of the National Health Council. We obtained the approval of the Research Ethics Committee of the ABC Medical School/Faculdade de Medicina do ABC, with CAAE: 73945017.0.0000.0082, and Opinion No. 2,275,101. All individuals who agreed to participate in the study will sign the free and informed consent form (FICF). The FICF is also available in audio and Braille versions. The results will be disseminated through peer-reviewed journal articles and conferences. This study is registered in the Brazilian Registry of Clinical Trials under the number RBR-9sm9dp.

## 1 Introduction

People with visual impairment (VI) have loss of vision that causes impact on their daily living activities. Synonymous of VI are: blindness, low vision, subnormal vision, and visual incapacity, although there are peculiarities among them.^[1]^

The prevalence and cause of blindness vary with region and time. Overall, in 1990 there were about 31.8 million blind people and 172 million people with VI. In 2010, the numbers increased to 32.4 million blind people and 191 million people with disabilities.^[1]^

The autonomic nervous system (ANS), through the sympathetic and parasympathetic divisions, provide dynamic adaptations in the body, moment to moment, according to internal changes and/or depending on alterations on the external environment.^[2]^ Since VI is an adverse condition, association with changes in systemic autonomic activity, such as heart rate variability (HRV), blood pressure (BP), changes in body temperature, and electrical conductivity of the skin, among others, is expected.^[3]^

Since cardiac function is regulated by the interaction of the sympathetic and parasympathetic branches of the ANS, HRV can be an important tool to investigate the oscillations in the intervals between consecutive heart beats (R-R intervals), which are related to the influences of the ANS on the sinus node, the natural heart pacemaker. It is a noninvasive technique, whose analysis can be performed using a linear method at time and frequency domains, or by nonlinear methods in the chaos domain^[4]^

Time domain methods use mathematically simple techniques to measure the variability present in the R-R intervals by calculating their mean and the variations of the standard deviation of heart rate (HR) over time.^[5,6]^ While frequency domain methods use spectral analysis that allows the decomposition of HR variation at a given time into its fundamental oscillatory components, that is, the time series is decomposed into different frequency components.^[7]^

In nonlinear analysis, the chaos theory approach considers systems, dynamic, deterministic, governed by nonlinear equations, and sensitive to initial conditions.^[8]^ This form of analysis may more adequately reflect changes in the autonomic modulation of biological systems, as evidence indicates that the mechanisms involved in cardiovascular regulation probably interact nonlinearly with each other.^[9]^

It is important to consider whether VI affects the cognitive and attitudes of the patients, as well as the clinical variations of the cardiac autonomic modulation, either if the low vision is acutely presented to the subject or chronically present, after a longer period of disability. In some diseases, not involving the visual apparatus, this interference in autonomic modulation may be reflected by a reduction in HRV, which has sometimes been suggestive of an indication of worse prognosis in cardiovascular diseases^[8,10]^ and related to the appearance of arrhythmias and sudden death.^[11,12]^

On the contrary, limited literature has been published about this subject, namely visual deficiency vs HRV, which strengthens and justifies the accomplishment of this research. Thereby, this study focused to analyze the blindness stress by monitoring the activity of the ANS in the heart in subjects submitted acutely to low vision and also in subjects with chronic visual deficiency.

## 2 Materials and methods

### 2.1 Type, location, and population of the study

**Figure 1:**
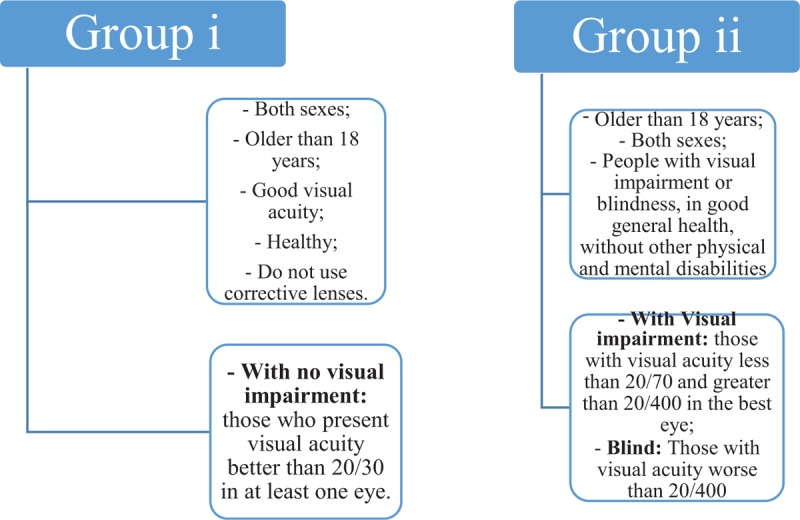
Subject eligibility criteria.

This study protocol follows the items of the Standard Protocol for Randomized Trials. It is an experimental study, specifically a randomized clinical trial on the autonomic HR modulation response, in which 50 volunteers with normal vision (Group i) and 50 individuals with visual deficiency (Group ii) have attended the Pedagogical Service of Support Center for People with VI (Fig. 1).

### 2.2 Subject eligibility criteria

People of both sexes, over 18 years old, with good visual acuity, healthy, and who do not need correctional lenses, such as glasses, will be eligible to form Group i. The Group ii will be constituted by people, also over 18 years of age, both genders, with VI or blindness, with good general health, and without other physical and mental disabilities. In this context, those who present visual acuity better than 20/30 in at least 1 eye will be admitted as participant of Group i, without VI. A 2nd group with VI (Group ii), will include individuals with visual acuity less than 20/70 and greater than 20/400 in the best eye; as blind people with visual acuity worse than 20/400, finger counts at 1 m and with lack of light perception in the best eye, according to criteria of the World Health Organization.^[13]^

Individuals who do not comply the clinical criteria described or refused to participate or continue in the survey will be excluded.

Volunteers will be instructed for not drinking alcohol and/or caffeine within 24 hours prior to the intervention.

### 2.3 Expected risks

Risks are minimal. If patients feel uncomfortable during evaluation sessions, they can withdraw the research at any time.

### 2.4 Expected benefits

Provide scientific information from comparative assessment between the groups, through the analysis of HRV during the proposed tasks.

Demonstrate ANS reactions in different situations with individuals without visual disorder and individuals with VI.

### 2.5 Intervention

Liao et al^[14]^ studied 2 groups with 20 patients each, performing a 30-minute exercise program of electric bicycle and a 5-minute cooling during the treatment of hemodialysis (HD) in the renal unit. The exercise was performed under supervision of a physician and a nurse specialized in rehabilitation. This protocol was adapted to be carried out with 2 groups of 50 people each. We also modified the time for the accomplishment of activities, they will be developed in 45 minutes, split in 3 periods of 15 minutes each. Times were defined as rest, intervention, and recovery. The execution will be supervised by a researcher, an ophthalmologist, a professional physical educator, a nurse, a student, and a secretary.

Marques et al^[15]^ measured exercise intensity by using the Borg rating of perceived exertion scale. In our study, patients will perform activities without load prescription. In the study by Raimundo et al,^[16]^ the subjects walked on a treadmill for aerobic exercises split into 5 minutes of warm-up and 25 minutes of exercise, reaching 50% to 70% of their maximal HR. Morais et al^[17]^ used in its protocol in patients in HD, aerobic exercise of cyclical movements of the lower limbs with 45% to 60% of intensity of its HR maximum.

Morais et al^[17]^ performed aerobic exercises with a total time of 30 minutes, 3 times a week for 3 months. The subjects were instructed to remain with arms extended normally in the extension of the chair next to the body, accommodated in the best possible way. The lower limbs that were the most active part in the exercise performed a constant rotation (pedals) throughout the aerobic exercise in the mechanical cycle ergometer for lower limbs. Patients will be encouraged to increase their intensity according to individual capacity, respecting the principle of biological individuality.

**Figure 2:**
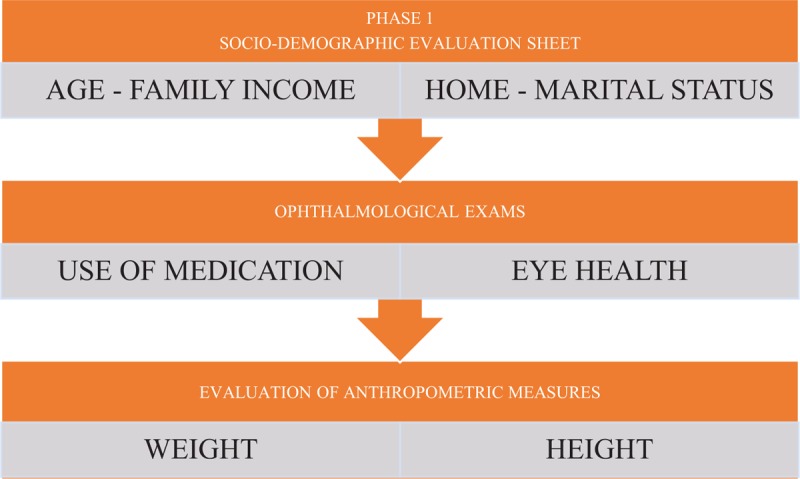
Phase 1 protocol steps.

**Figure 3:**
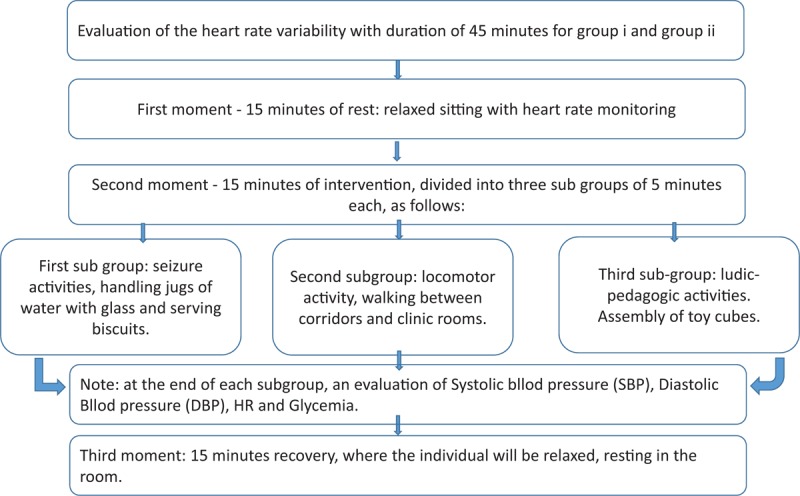
Flowchart of phase 2 protocol steps.

In our case, in the intervention phase, the activities performed will be sensorial, by handling objects, the motor part consists in walking blindfolded for 5 minutes through the corridors and clinic rooms, and at the end cognitive activities will be performed with pedagogic games. The protocol was modified so that individuals from both groups will be monitored for 45 minutes, divided into 3 times of 15 minutes each. Each phase will be monitored for its HRV and will be called rest-intervention-recovery. The phase with greater intensity will be the intervention, because the time of this phase will be divided in 3 periods of 5 minutes each. In each subphase of 5 minutes, the individuals will perform 3 different activities. The 1st task will be to serve glasses with water and food. Then, the next task will be locomotion through the clinic areas and the last stage activities will be to assembly pedagogical toys (Figs. 2 and 3).

### 2.6 Team work

**Figure 4:**
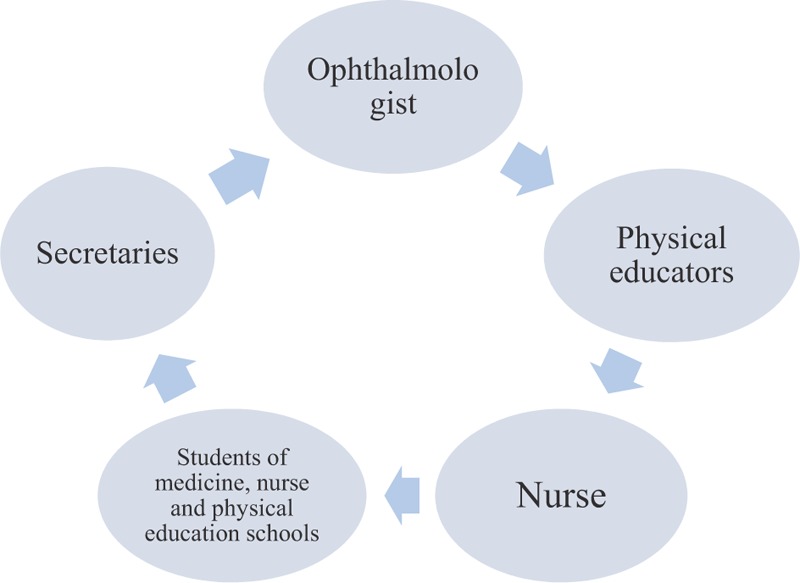
Team work.

The research team will include 2 ophthalmologists, 2 physical educators, a nurse, 2 medical, nurse and physical education academics, and 2 secretaries from the Rio Branco Eye Health Center (Fig. 4).

### 2.7 Data collection instruments

*First phase*: A sheet will be used to collect personal and sociodemographic data, complemented with clinical data according to the execution of medical examinations and interventions in the study participants.

Initially, it is intended to form the Group ii by searching people with VI from the Pedagogical of Support Center CAP-AC database, a public institution that exclusively provides socioeducational support services to low vision patients and the Centro de Saude Ocular (CSO), which is a private clinic of ophthalmologic care in Rio Branco City. Individuals for Group i those without VI who have good visual acuity will be invited from the CSO, seeking to match them as closely as possible with Group ii, regarding gender, age, occupation, etc.

After initial selection, volunteers will be submitted to routine clinical-ophthalmological examination, where aspects of general health will be investigated, as well as medication that may interfere with ANS, among others. Other aspects of eye health, such as visual acuity, corrective lenses, and history of ophthalmic surgery also will be evaluated.

Clinical and ophthalmological examinations that will indicate the study independent variables will be performed at the CSO, which has qualified human resources and medical equipment for anamnesis, visual acuity measurement, ocular pressure measurement, among others, with no costs for participants.

**Figure 5:**
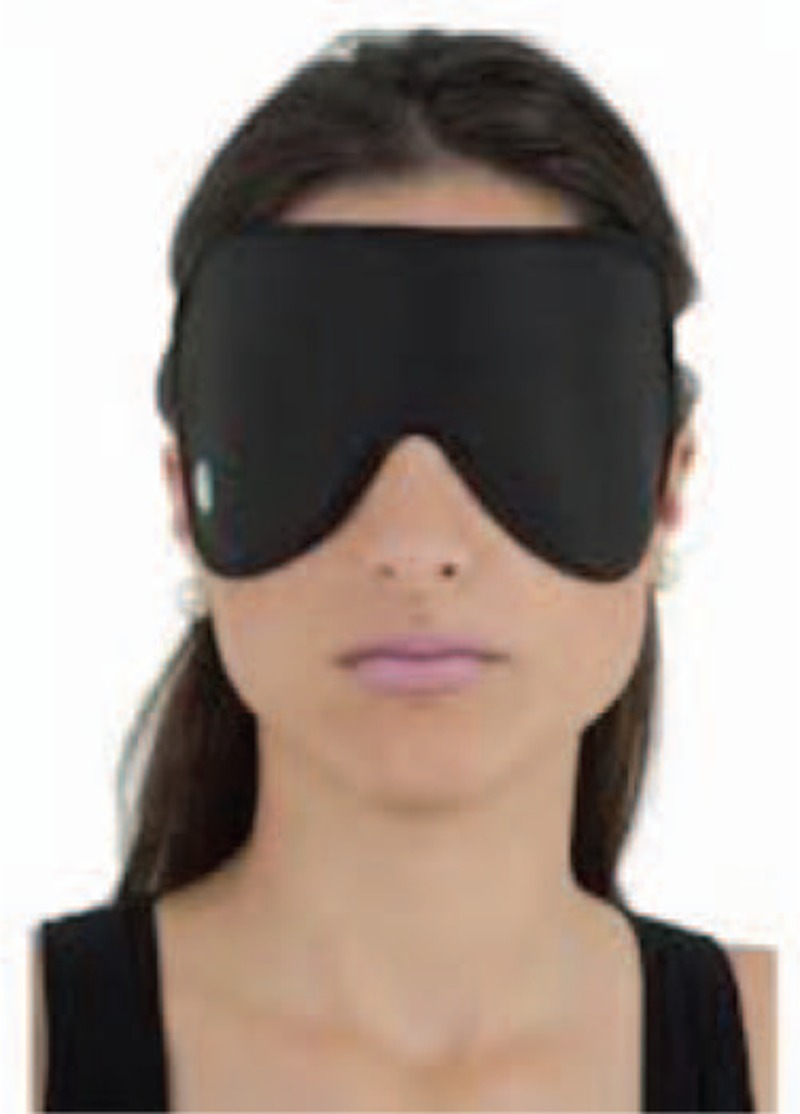
Maximum occlusion.

*Second phase*: In this phase, data of HRV will be collected. The protocol will be the same for both groups (Group i and Group ii). It will be divided in 3 moments of 15 minutes each: rest-intervention-recovery. After taking anthropometric measures, the participant of Group i will be given a brief rest in seated position, in a quiet environment, then the systolic, diastolic, and mean BP and HR will be measured to define a baseline condition (rest phase). Then, in the 2nd step or intervention phase, both eyes of the participant will be blindfolded with a sleeping mask that provides maximum occlusion (Fig. 5), remaining with vigilance and companionship.

**Figure 6:**
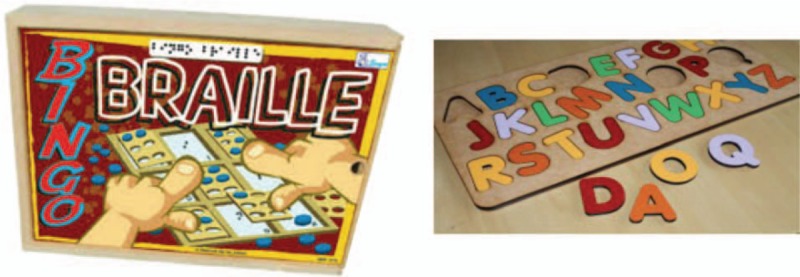
Educational toys.

Intervention phase will be subdivided into 3 times of 5 minutes each to perform 3 different tasks, as follows:

1. First task, the participant still blindfolded will be asked to perform seizure tasks, serving water and/or biscuits on a tray, sitting with the objects placed at the table in front of him.
2. Second task is for motor locomotion, such as walking through clinic areas between corridors and rooms.
3. Third task will be ludic-pedagogical activities as fitting of different objects according to their mold like a puzzle (Fig. 6).

At each stage of 5-minute intervention activities, the patient will be monitored for their systolic and diastolic HR and level of blood glucose.

In the 3rd phase called recovery with a duration of 15 minutes, the participants will sit relaxed and resting, continuing to monitor the collection of autonomic cardiac activity through the HRV.

Participants of Group ii, after verifying their anthropometric measures, will be submitted to the same protocol of Group i.

Measurements include systolic blood pressure, DBP, mean arterial pressure, HR, and blood glucose level.

**Figure 7:**
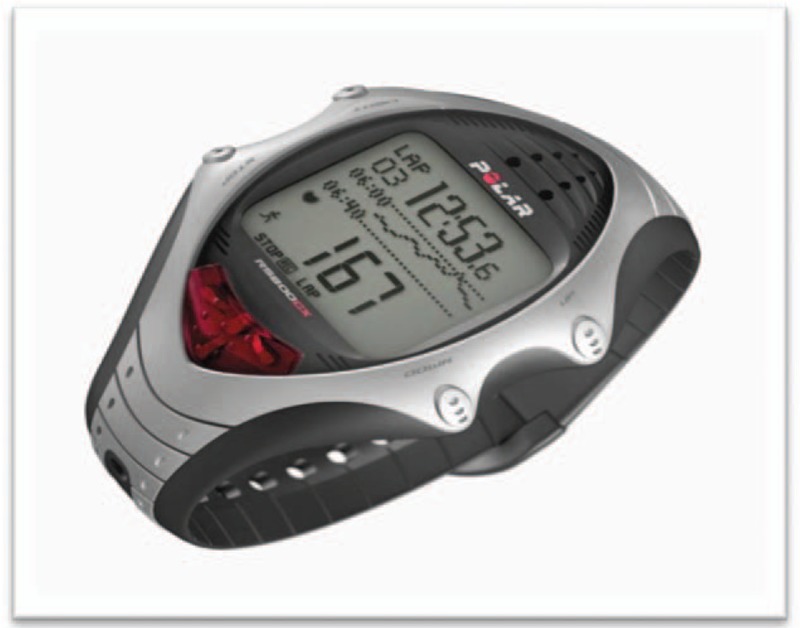
RS800CX, Polar Electro, Finland.

**Figure 8:**
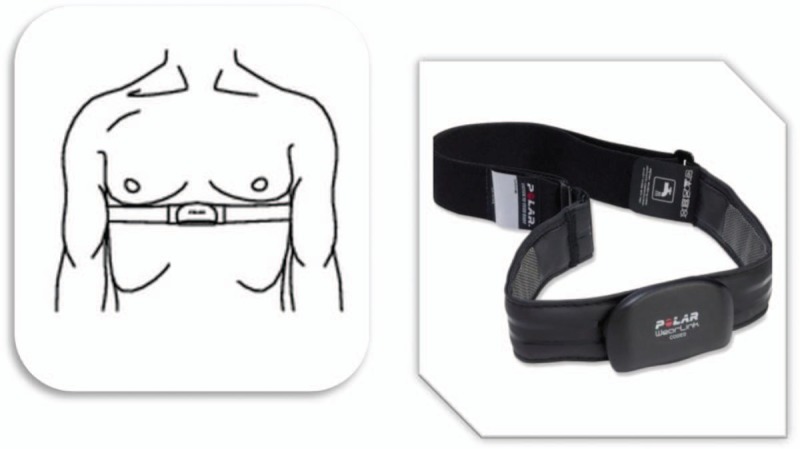
Placement of the belt on the patient.

The HR monitoring for further analysis of HRV will be obtained by an HR receptor RS800CX, Polar Electro, Finland. This device was validated to locate the heart beat for HRV analysis (Fig. 7). At the appropriate moment for collection during the estimated 45 minutes of evaluation, the strap will be placed on the chest of volunteers and the HR receptor (Fig. 8), which is an equipment previously validated for HR reception, beat and use these data for HRV analysis. The receiver temporarily stores the data that will then be processed on computers.

**Figure 9:**
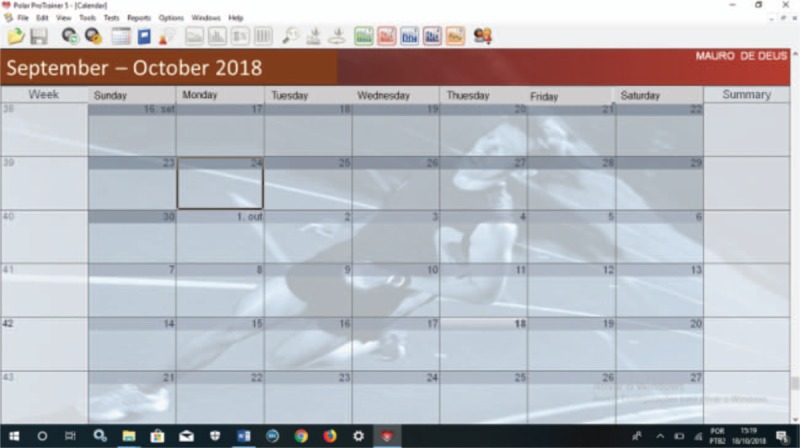
Data transfer.

The recorded pulses will be directed to a computer through an infrared signal emitting interface for analysis of the HRV by Polar Precision Performance software. For data analysis, after storage in the software, they will be filtered at a moderate intensity for the elimination of ectopic beats and/or noises. The HRV analysis will be performed in time and frequency domain (linear analysis) and also by nonlinear analysis (Fig. 9).

Data collection of intervention in subjects of Group i, temporary deprived of vision, will be performed individually, on working days from 8:00 to 12:00 am, recorded in a standardized sheet (Annex 4). The venues will be discrete and isolated, which could be in the outpatient or recreation/training environment of CAP-AC and the CSO in Rio Branco.

### 2.8 Data analysis

Data will be analyzed using the Statistical Package for Social Science, version 22.0. Descriptive analysis will be performed for all variables. Data will be expressed as mean ± standard deviation or median (interquartile range), where appropriate. Sample distribution will be tested with the Shapiro–Wilk test. For comparison of initial and final values between groups, the unpaired Student *t* test for parametric distributions and the Mann–Whitney test for nonparametric distributions will be applied. Statistical significance will be considered at the level of *P* < .05 (or 5%).

As a risk measure, odds ratio will be calculated, adjusted, and not adjusted with their respective 95% confidence intervals. The odds ratio = 1 of lowest risk for the outcome of interest will be considered as the base category for each independent variable.

**Figure 10:**
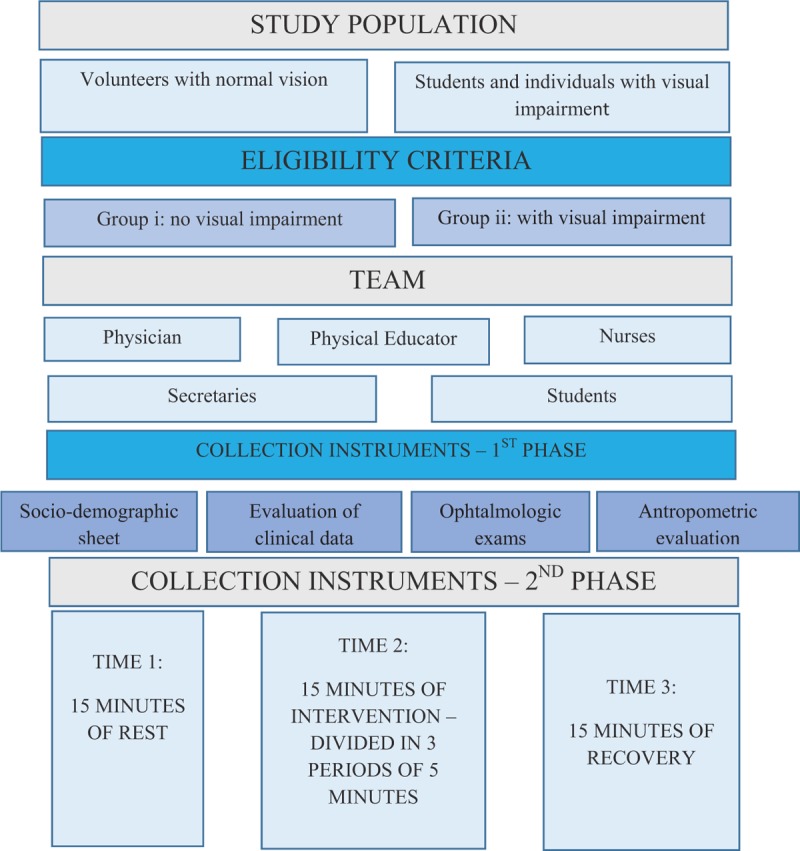
General protocol flowchart.

Statistically significant variables in the univariate analysis will compose the model for the multivariate analysis. In this multivariate analysis, multiple logistic regression will be used (Fig. 10).

## 3 Discussion

Overall, in 2010, out of a total of 32.4 million blind and 191 million visually impaired, 0.8 million were blind and 3.7 million were visually impaired because of diabetic retinopathy (DR), with an alarming increase of 27% and 64%, respectively, in the 2 decades from 1990 to 2010. These numbers were lower in regions with younger populations (<2% in Southeast Asia and Oceania) than in high-income regions (North America, Western Europe, and Asia) with populations relatively aged (>4%).^[18]^

Dyslipidemia and hypertension may also influence DR, although in the context of individual patients, the associations between plasma lipids, lipoproteins, and DR are not strong enough to define the risk of retinopathy. Similarly, hypertension has been associated with increased risk of RD, and some data indicate that patients may benefit from the use of antihypertensive agents. However, recent studies have shown that more intensive BP monitoring does not confer additional benefits on the progression of retinopathy compared to standard follow-up. Taken together, the optimization of systemic risk factors is clearly important; however, even hyperglycemia (as measured by HbA1c) may represent only about 10% of risk of DR, and combined hypertension and dyslipidemia can lead to <10% risk in some cohorts. Such data strongly suggest that additional unidentified factors also play critical roles in the initiation and progression of DR.^[19]^

Universality is linked to the guarantee of the right to the same level of healthcare for all Brazilians, without any discrimination. It is very relevant researching about better ways of achieving positive results, new knowledge, and products that will improve the health of the population, either with a new diagnosis or new therapeutic treatments, or even with actions aimed at promoting the health of the population.^[20,21]^ The authors intend to propose a good quality protocol in its execution, having patient safety and demonstrating reliable information for healthcare area, contributing for better health care indicators of visually impaired people.

### 3.1 Declaration, ethics approval, and consent to participate

This study will follow the guidelines that regulate human research in Resolution No. 466/12 of the National Health Council. We obtained approval from the Research Ethics Committee of Faculdade de Medicina do ABC, with CAAE: 73945017.0.0000.0082, and Opinion No. 2,275,101. All patients who agree to participate in the study will sign an informed consent form (FICF). The FICF is also available in audio and Braille versions. This study is registered in the Brazilian Registry of Clinical Trials under the number RBR-9sm9dp.

Temporary deprivation of vision pretends to simulate a fleeting “acute blindness” during 45 minutes to study the behavior of the ANS acting in the human heart, without significant risks to the participants and without waiting for natural events of sudden loss of bilateral vision.

Information and data collected will remain available to the participants in the archives of the institutions involved in the research, during and after the closure of this study.

## Author contributions

**Fabiano Santana de Oliveira:** collected data, performed conduction of experiments, and draft the manuscript.

**Italla MP Bezerra:** garner performed statistical analysis, draft the manuscript, extensively reviewed the manuscript, English grammar, and spelling.

**Luiz Carlos de Abreu:** garner performed statistical analysis, draft the manuscript, extensively reviewed the manuscript, English grammar, and spelling.

**Mauro José de Deus Morais:** collected data, performed conduction of experiments, and draft the manuscript.

**Monica A Sato:** supervised the study, draft the manuscript, wrote introduction and discussion section, and gave final approval for the version submitted for publication.

**Renaldo D Moreno:** collected data, performed conduction of experiments, and draft the manuscript.

**Vitor E. Valenti:** supervised the study, draft the manuscript, wrote introduction and discussion section, and gave final approval for the version submitted for publication.
